# Chemical Composition and In Vitro Antiplasmodial Activity of the Ethanolic Extract of *Cyperus articulatus* var. *nodosus* Residue

**DOI:** 10.3390/pathogens9110889

**Published:** 2020-10-27

**Authors:** Francisco Flávio Vieira de Assis, Nazaré Carneiro da Silva, Waldiney Pires Moraes, Lauro Euclides Soares Barata, Antonio Humberto Hamad Minervino

**Affiliations:** 1 Laboratory of Animal Health, LARSANA, Federal University of Western Pará, UFOPA, Santarém, Pará 68040-255, Brazil; flavioassis131995@hotmail.com; 2 Pharmacology Laboratory, Federal University of Western Pará, Santarém, Pará 68040-255, Brazil; nazaresilvaufopa@hotmail.com (N.C.d.S.); waldineypires@gmail.com (W.P.M.); 3 Laboratory for Research & Development of Bioactive Products, Federal University of Western Pará, Santarém, Pará 68040-255, Brazil; lauroesbarata@gmail.com

**Keywords:** malaria, priprioca, phytotherapic, antimalarial

## Abstract

*Cyperus articulatus* L. is popularly known as priprioca. Its rhizomes are used as a medicine in the treatment of malaria in traditional medicine. Since priprioca oil is extracted for commercial purpose, we evaluated if the components from the priprioca residue can be a source of antiplasmodial active molecules. This study aimed to determine the in vitro antiplasmodial and cytotoxicity activities of the ethanolic extract of *C. articulatus* as an in vitro antiplasmodial agent. From the solid residue of the plant rhizomes, 40 g samples were removed and subjected to hot extraction using a Soxhlet extractor. The in vitro antiplasmodial activity was determined using the W2 and 3D7 strains of *P. falciparum*. The phytochemical study identified the following main compounds: corymbolone (14.25%), cyclocolorenone (9.75%), and cadalene (8.36%). The extract exhibited moderate IC_50_ (inhibitory concentration) against the two strains of *P. falciparum*: 1.21 ± 0.01 against the W2 strain and 1.10 ± 0.06 µg/mL against the 3D7 strain. Our results show the therapeutic potential of priprioca residue as a low-cost antiplasmodial agent.

## 1. Introduction

Malaria is an infectious, parasitic disease that affects millions of people in the tropical and subtropical regions worldwide, mainly in Africa, Southeast Asia, and the Amazon region of South America [1,2,3]. According to the World Health Organization [4], there were 228 million cases of malaria worldwide in 2018 with 405,000 deaths. Malaria is considered the most important potentially fatal parasitic disease of human beings [5].

In Brazil, most cases are in the Amazon region, owing to, among other factors, the favorable conditions for the existence of the vector, the lack of adequate sanitation, potential risk of zoonotic transmission from nonhuman primates, and disorderly settlements near too forested areas, causing serious problems in public health and great economic impact affecting millions of people [6,7,8].

Malaria, a disease caused by protozoa belonging to *Plasmodium*, has a large impact on the world, putting at risk approximately 40% of the world population (about 2.4 billion people) in more than 100 countries [9]. This disease is transmitted to humans by infected female *Anopheles* mosquitoes. *P. falciparum* is the most dangerous species of the genus because it can lead to the most severe form of malaria, cerebral malaria, which without treatment is invariably fatal and in patients treated adequately the mortality rate is around 15–20% [10,11].

The resistance of *P. falciparum* to artemisinin derivatives in Southeast Asia threatens malaria control [12,13]. High prevalence of *P. vivax* genotypes associated with chloroquine resistance highlights the severe situation of Malaria treatment in Oceania and South Asian regions [14]. The search for new drugs for treatment is steadily increasing because of the parasite’s resistance to traditional therapy [15].

The importance of natural products, including those derived from plants, is recognized in the development of therapeutic drugs for different purposes in animals [16] and humans. Approximately 40% of the drugs currently available have been developed directly or indirectly from natural sources, of which 25% are from plants [17,18]. There is a great diversity of plant species worldwide and in the Brazilian Amazon, with the potential for investigation of new secondary metabolites with antiplasmodial action [6,19]. *Cyperus articulatus* L. belongs to the *Cyperaceae* family and is popularly known as priprioca in Brazil, mainly in the Amazon region, where it is traditionally used as a sedative, anticonvulsant, and anti-inflammatory agent [20]. Its rhizomes are used as a source of herbal medicines for fever and seizures and in the treatment of malaria in traditional medicine [21]. There has been a growing interest in studies of *C. articulatus* in recent years owing to the pleasant aroma of the volatile oil obtained from the rhizome of the plant for perfumery industry [20] and its medicinal effects.

When essential oil is removed from priprioca rhizome, organic solid waste is generated which still contains significant amounts of metabolites with medicinal activity. In this context, the objective of the present study was to assess the effect of the ethanolic extract of *C. articulatus* rhizome residue as an in vitro antiplasmodial agent.

## 2. Material and Methods

### 2.1. Collection and Identification of Plant Materials

Rhizomes of the species *Cyperus articulatus* var. *nodosus,* popularly known as priprioca, were collected in August 2014, in the Tabocal region of the municipality of Santarém, Pará, Brazil (−54°43’00.10” W −02°37’41.10” S). The species was identified by the botanist Dr. Antônio Elielson Sousa da Rocha, from the Museu Paraense Emilio Goeldi, where a specimen is deposited (MG-207174). The plant’s name was verified on the online website (www.theplantlist.org) of the Royal Botanic Gardens, Kew, accessed on July 7, 2020.

### 2.2. Preparation of the Ethanolic Extract of C. articulatus (EECA)

Five kilograms of the rhizomes of *C. articulatus* were collected. After collection, the rhizomes were cleaned, dried for three consecutive days, and ground in a knife mill in an open environment. After drying, the crushed material was subjected to hydrodistillation by a 150 L steam distillation system for 1.5 to 4 h, to obtain the essential oil. From the solid vegetable residue waste originating from the extraction by steam system, 40 g samples were removed and subjected to hot extraction in a Soxhlet extractor using ethanol 96%. The solvent of the solution resulting from the hot extraction was evaporated in a rotary evaporator under reduced pressure to obtain a concentrate of the ethanolic extract of the residue of *C. articulatus*.

### 2.3. Chromatographic Analysis of the EECA

The analysis of the chemical composition of the EECA was performed in an Agilent gas chromatograph, model HP-6890 equipped with a selective Agilent mass detector, model HP-5975 using an HP-5MS capillary column (30 m × 0.25 mm × 0.25 µm) under the following conditions: injector temperature = 250 °C, column = 80 °C, heating rate = 5 °C/min to 280 °C (20 min), and detector = 300 °C. Helium was used as carrier gas at a flow rate of 1 mL/min and a selective mass detector operating at 70 eV, m/z = 30 to 500 u.m.a. The EECA was solubilized in ethyl acetate at a concentration of 20 mg/mL and the identification of the major compounds in the extract was performed by comparison with the electronic library of the equipment (NIST-11).

### 2.4. Assessment of Cell Viability

For the analysis of cell viability, 3-(4,5-dimethylthiazol-2-yl)-2,5-diphenyltetrazolium bromide (MTT, Sigma-Aldrich, Inc. St. Louis, MO, USA) was used with the cell line WI-26VA-4 (lung fibroblast ATCC CCL-95.1); this strain is part of the animal cell bank of the Cell Biology Service (SBC) of the Ezequiel Dias Foundation (FUNED). The cells were maintained in RPMI1640 medium supplemented with 21 mM bicarbonate, 40 mg/mL gentamicin, and 10% (*v*/*v*) inactivated fetal bovine serum. A cell suspension was distributed in a 96-well plate at a concentration of 1 × 10^5^ cells/well, and incubated for 24 h at 37 °C in a humid atmosphere containing 5% CO_2_. After that, the cells were washed with phosphate-buffered saline. Subsequently, the EECA was added at different concentrations, ranging from 1000 to 0.1 μg/mL. After 48 h of incubation, the plate was washed again with phosphate-buffered saline with 100 μL of MTT tetrazolium salt added at a concentration of 5 mg/mL. The plate was incubated again for another 3 h. After discarding the supernatant, 50 μL of dimethyl sulfoxide (DMSO, Sigma-Aldrich, Inc. St. Louis, MO, USA)) was added, and the color obtained was evaluated by reading on a microplate spectrophotometer (model Espectramax M5e, Molecular Devices LLC, San Jose, CA, USA) at a wavelength of 550 nm. The concentration that inhibited cell growth by 50% (LC_50_) was determined in comparison to cells cultured without the test compound.

### 2.5. Evaluation of Hemolytic Activity

Fresh blood (5 mL) was collected in an EDTA tube and centrifuged at 1000× *g* for 5 min at 4 °C. The supernatant was removed, and the pellet with red blood cells was washed five times with saline solution (0.9% sterile) and then suspended in the same solution to obtain a 1% (*v/v*) red cell solution. The test compound was diluted in a 1% DMSO solution and tested in triplicate at concentrations of 1000 to 15.6 μg/mL in seven serial dilutions (1:2), and 100 μL of the 1% red blood cell suspension was added (*v/v*) in each well. The resulting suspensions were incubated for 60 min at 37 °C. After incubation, the samples were centrifuged for 2 min at 1000× *g*. The supernatants were transferred to 96-well plates and the release of hemoglobin was measured by absorbance at 450 nm (Abs450nm) using a BioTek Synergy HT multiplate reader. To control 100% hemolysis, red blood cells were suspended in 1% (*v/v*) Triton X-100. As a control without hemolysis, a 0.9% saline solution was used. The percentage of hemolysis was determined using the following equation: [(Abs450nm treated sample-Abs450nm untreated)/(Abs450nm1% Triton X-100-Abs450nm untreated)] × 100 [22].

### 2.6. Evaluation of In Vitro Antiplasmodial Activity

In vitro antiplasmodial activity was determined using W2 (chloroquine-resistant) and 3D7 (chloroquine-sensitive) strains of *Plasmodium falciparum* grown in human A+ red blood cells in a predominantly ring stage, synchronized using the sorbitol method [23]. RPMI 1640 culture medium supplemented with 25 mM HEPES, 21 mM sodium bicarbonate, 300 mM hypoxanthine, 11 mM glucose, 40 mg/mL gentamicin, and 10% (*v*/*v*) inactivated human plasma was used. The cultures synchronized with 2% parasitemia and 2% hematocrit were distributed in a 96-well microplate. The EECA was added at a starting concentration of 50 μg/mL and twofold serial diluted in incomplete culture medium and 4% DMSO until the final concentration of 0.049 μg/mL. We tested the following concentrations of ECA: 50, 25, 12.5, 6.25, 3.125, 1.56, 0.78, 0.39, 0.195, 0.098, 0.049 μg/mL. Chloroquine was used as a standard antimalarial. The plate was incubated for 48 h at 37 °C with 5% CO_2_ and 95% relative humidity. Smears corresponding to each well were made. After being fixed with methanol and stained with Giemsa, the smears were used to count the parasitemia under an optical microscope (1000×). The activity of the compounds was expressed as the percentage of reduction in parasitemia in relation to the negative control (without any antiplasmodial drug), which was considered as 100% of the growth of the parasite. The experiment was performed in triplicate with each concentration of EECA extract, positive and negative controls, tested three times, totaling 39 independent assays per strain (total of 78). The slide reading was blinded (i.e., the slides had codes, and the observer did not know the treatment used in each slide) and performed by the same trained researcher. The concentration of extract that inhibited 50% of the growth of the parasite (IC_50_) was determined through dose–response curves, as a function of nonlinear regression.

### 2.7. Selectivity Index

The selectivity index (SI) is the relationship between the cytotoxic and antiparasitic activities of each compound. The SI was obtained from the ratio between the LC_50_ value for WI cell line and the IC_50_ for *P. falciparum* (strain W2) according to the formula: SI = LC_50_ cell line WI/IC_50_ *P. falciparum* (strain W2). Values greater than 10 were considered indicative of high selectivity, whereas substances with values below 10 were considered to have low selectivity [24].

### 2.8. Statistical Analysis

Statistical analyses were performed using OriginPro 8.0 (OriginLab Corporation, Northampton, MA, USA) to determine the IC_50_ value of the in vitro test.

## 3. Results

The phytochemical study of the ethanolic extract of *C. articulatus* obtained from residues derived from the extraction of essential oil resulted in the identification of the following main compounds: alpha-bulnesene, cadalene, cyperotundone, cis-thujopsenal, cyclocolorenone, corymbolone, hexadecanoic acid ethyl ester, 9,12-octadecanoic acid ethyl ester, 9-octadecenoic acid ethyl ester, and cholesta-3,5-diene. Together, these compounds represented more than 60% of the total sample (Table 1).

The compound identification shows the presence of mainly sesquiterpenes, wherein the highest yield (14.25%) was obtained for corymbolone. The EECA at the tested concentrations did not show hemolysis, similar to the control with 0.9% saline.

In the in vitro antiplasmodial test, the EECA presented an IC_50_ value of 1.21 ± 0.01 obtained from dose–response curves for the culture of *P. falciparum* against the W2 strain (chloroquine-resistant) and an IC_50_ value of 1.10 ± 0.06 against the 3D7 strain (chloroquine-sensitive) (Table 2). In the cytotoxicity test with the WI cell line, using the MTT colorimetric method, the EECA showed an LC_50_ value of > 100. In calculating the SI based on the ratio between the toxic dose and its antimalarial activity, *P. falciparum* demonstrated that the EECA is a safe compound with SI values > 91 for the 3D7 strain and 83 for the strain W2 (Table 2).

## 4. Discussion

Preliminary studies evaluating the extract of *C. articulatus* also identified elevated concentration of corymbolone among all the other identified substances and showed in vitro antiplasmodial activity [25]. Considering the verified antiplasmodial action and the low toxicity of this molecule, it may has potential to be used as an alternative of chloroquine, which has adverse effects [26,27]. Alpha-bulnesene has biological activity related to the inhibition of platelet activation factor [28]. Toxicity analysis, which is necessary when investigating the biological activity of products with potential for phytotherapy, is conducted with the objective of determining the potential of new substances and products without causing risk to human health and classifying them according to their potential for lethality or toxicity as established by legislation [29].

Research using ethanol extract from leaves of *Montrichardia linifera* (Arruda) Schott showed moderate antiplasmodial activity (10 < IC_50_ < 100 μg/mL) [30], which is below that found in this study (IC_50_ = 1.21 µg/mL). However, work with *Artemisia turcomanica* extract obtained antiplasmodial activity with IC_50_ and IC90 values of 0.90 ± 0.27 mg/mL and 1.62 ± 0.68 mg/mL, respectively [31].

As for cytotoxic activity, one study found that *C. articulatus* essential oil was moderately cytotoxic to monkey kidney cells [32]. However, with *C. articulatus* essential oil, a noncytotoxic result was obtained for human pulmonary fibroblasts by cell viability assay with MTT (LC_50_ > 100 μg/mL) [32]. It was also observed that the IC_50_ of W2 and 3D7 strains of *P. falciparum* treated with essential oil were 1.21 μg/mL and 2.30 μg/mL, respectively [33], consistent with the findings in this study. 

In addition, an in silico study of the activities of triterpenes and iridoids isolated from *Himatanthus articulatus* (Vahl) Woodson did not identify any molecules with antiplasmodial activity [34]. One limitation of the present study is related to the source of antiplasmodial activity. We found three major components in the residue extract, corymbolone (14.25%), cyclocolorenone (9.75%), and cadalene (8.36%). The antimalarial activity is most likely from one of those or from a combined effect between two or more compounds present in the tested extract. Further studies are required to verify the antiplasmodial activity using purified chemical components found in the ethanolic extract of priprioca residue, which could be used as a new drug for malaria treatment.

## 5. Conclusions

The ethanolic extract obtained from the rhizome residue of *Cyperus articulatus* from Santarém, Brazil, contained corymbolone as the main compound identified and exhibited a moderate level of activity against two strains of *Plasmodium falciparum* (W2 and 3D7). The EECA also showed low cytotoxicity against the human cell line WI-26-VA-4. Our results demonstrate the therapeutic potential of *C. articulatus* as an antiplasmodial agent.

## Figures and Tables

**Table 1:** Chemical composition of the ethanolic extract of the *Cyperus articulatus* residue.

Identification	t_R_ (min)	Rel%
alpha-bulnesene	15.01	1.18
cadalene	18.95	8.36
cyperotundone	19.34	2.88
cis-thujopsenal	19.59	4.19
cyclocolorenone	20.49	9.75
corymbolone	23.32	14.25
hexadecanoic acid ethyl ester	25.21	5.99
9,12-octadecadienic acid ethyl ester	28.27	3.24
9-octadecenoic acid ethyl ester	28.38	5.5
cholesta-3,5-diene	39.63	4.82
Total identified:		60.16

t_R_: retention time; Rel%: relative percentage.

**Table 2:** In vitro antiplasmodial activity, cytotoxicity (human lung fibroblast, Wi 26VA-4), and selectivity index (SI) of ethanolic extract of *Cyperus articulatus* rhizomes against *Plasmodium falciparum* strains W2 and 3D7, and using chloroquine as control.

Compound	IC_50_ (µg/mL) ± SD *		Antiplasmodial Activity	LC_50_ (µg)	SI	
	3D7	W2			3D7	W2
Ethanolic Extract	1.10 ± 0.06	1.21 ± 0.01	Active	> 100	> 91	83
Chloroquine	0.46 ± 0.08	0.21 ± 0.01	Active	> 100	> 200	> 200

IC_50_: average inhibitory concentration; LC_50_: mean lethal concentration. * Values are expressed by mean ± SD (standard deviation).
